# Phytochemical cocktail of *Asanadi gana*extract in the management of diabetes

**DOI:** 10.6026/97320630019299

**Published:** 2023-03-31

**Authors:** Rekha Kumari Dulala, Manikandan Balraj, Sangeeta Chandrashekar, Muninathan N, Nithiya Rajapandiyan, Ramya Badrachalam, Vadivel Mani

**Affiliations:** 1 Department of Biochemistry, Konaseema Institute of Medical sciences and research foundation, Amalapuram, East Godavari Dt-533201, Andhra Pradesh, India; 2 Department of Physiology, Konaseema Institute of Medical sciences and research foundation, Amalapuram, East Godavari Dt-533201, Andhra Pradesh, India; 3 Department of Physiology, Bharath Institute of Higher Education and Research, Chennai-600073, Tamilnadu, India; 4 Central Research Laboratory, Meenakshi Medical College and Hospital, Kanchipuram-631552, Tamilnadu, India; 5 Department of Physiology, Arunai Medical College & Hospital, Tiruvanamallai-606603, Tamilnadu, India; 6 Department of Biochemistry, Sri Manakula Vinayagar Medical College and Hospital, Puducherry - 605107, Tamil Nadu, India

**Keywords:** Asanadi gana, Antioxidant, Organic radicals scavenging activity

## Abstract

It is of interest to investigate that the phytochemical analysis, *in-vitro *antioxidant potential and glycosidase inhibitory potential of *Asanadi gana*a polyherbal formulation. *Asanadi gana*is a classical Ayurvedic polyherbal formulation, markedly used for alleviation of *Prameha* and *Medodosha*, which correlates in many ways with obesity, metabolic syndrome, and diabetes mellitus (madhumeha). The phytochemical constituents, total phenolic, total flavonoids, total tannin content, total antioxidant capacity, total reducing power, and free radical scavenging activity of the polyherbal formulation extracts were determined. Comparing it to the common medication Acarbose, its inhibitory impact against the digestive enzymes α-amylase and α-glucosidase was also examined. The formulation showed the presence of major constituents such as terpenoids, triterpenoids, sterols, flavonoids, tannins, phenolic, saponins, alkaloids and Glycosides. The ethanol extract had high phenolic content and flavonoid content, whereas the aqueous extract had more tannin content (181 ± 5.5µg/mg), (132 ± 5.50 µg/mg), (22± 1.6 µg/mg respectively. we conclude that the extracts of ayurvedic polyherbal formulations, particularly ethanol extract are a potential source of natural antioxidants and remarkable glycosidase inhibitory activity. Hence, *Asanadi gana*has the potential to be a safe and effective natural treatment for the delay or prevention of diabetic complications.

## Background:

Inflammatory burden and oxidative burden both are causing pathological factors to diabetes as well as complications of diabetes [1]. To protect against from oxidative burden the cells have a fabulous antagonist system like enzymatic and non- enzymatic antioxidants [2]. However, oxidative stress in diabetes compromise antioxidant leading to accumulation of reactive oxygen specious (ROS) and reactive nitrogen specious (RNS) this can provoke inflammation [3]. The inflammation and oxidative stress both are collision mechanisms cause extensive damage to biomolecules in the tissues; it is a global pressure to treat complications of diabetes in addition to insulin resistance [4]. The natural products are better choice compare to synthetic one, natural products less cost effect and advise effect [5]. According to a report from higher authority of health care more than 80% population in developing countries still is using ancient medicines for their primary health care [6]. Globally its awarded traditional medicine is safe to treat diseases [7], Ayurveda is one of Indian traditional medicine literal meaning of Ayurveda is "science of longevity", In the Ayurvedic literature "Sarangdhar Samhita" has highlighted the concept of poly-herbalism [8]. The fact of *Asanadi gana*polyherbal formulation is utilized in ayurvedic clinical practice for the treatment of obesity, skin diseases and conjointly with the management of diabetes [9,10]. However, there's a scarcity of scientific proof for the same. This current study was undertaken to gauge the antioxidant Potential and α-glycosidase enzyme repressive activity of a polyherbal formulation by victimization totally different standard tests. This herbal formulation prepared and marketed under the name of "*Asanadi gana*churoonam" Sri Dharamasthala Manjunatheswara Institute of Ayurveda & Hospital,Udupi, Karnataka, India.

## Materials & Methods:

## Drug and Chemicals:

Asanadi kwatha churoonam ayurvedic poly herbal formulation is prepared as per ayurvedic formulation procedure as in *Ashtanga Hrudayam & Sutrasthana* and purchased from Sri Dharamasthala Manjunatheswara Institute of Ayurveda & Hospital. The chemicals used for the study were all analytical grade. All solvents and chemicals (analytical grade) were purchased from Merck, SRL, Himedia, India. DPPH and TPTZ were acquired from Sigma-Aldrich, India.

## Herbal Concentrate Preparation:

About 25gm of dry Asanadi kwatha churoonam, ground to a rough powder, were gauged and macerated with 75 ml of solvents like acetone, ethyl acetate, ethanol and water, which were separately aliquoted and kept overnight in shaker. The extract was gathered after filtration using Whatmann No.1 filter paper and stored. 75 ml of solvent was added to the residual mixture and incubated in shaker for 24 hours and the extracts were gathered again using a Whatmann No.1 filter paper. This series of steps was duplicated again and the extracts were evaporated below 40°C.

## Phytochemical analysis:

Phytochemical are chemical compounds produced by plants, which generally help them to thrive combatant enemy, predators, or pathogens [11]. Phytochemical analysis of Acetone, Ethyl acetate, Ethanol and aqueous extract of Polyherbal formulation *Asanadi gana*revealed the presence of steroids, terpenoids, carbohydrates, Sterols, tannins, flavonoids, Proteins, saponins, alkaloids, organic acid, glycosides by their specific respective tests according to standard methods of phytochemical analysis [12,13], determination of total phenolic content, total flavonoid content, total Tannin content, total antioxidant capacity, Total Reducing Power, Radical Scavenging Activity and *in-vitro *anti diabetic activity like αl-amylase activity and α-glucosidase activity were measured by using standard methods.

## Statistical analysis:

All the data are expressed as the mean ± the standard deviation (SD). A one-way ANOVA test was performed to determine the significance of test samples compared to the controls and a value of p<0.05 was considered as significant.

## Result and Discussion:

## Preliminary Phytochemical analysis:

The Phytochemical investigation is a preliminary defend tool to identify the secondary metabolites. In the present study, several phytochemical constituents such as Flavonoids, Tannins and phenolic, Saponins, Terpenoids were present in various solvents extract like Acetone, ethyl acetate, Ethanol, and aqueous extracts shown (Table 1).

## Phytochemical evaluation:

## Total phenolic content:

**Figure 1:**
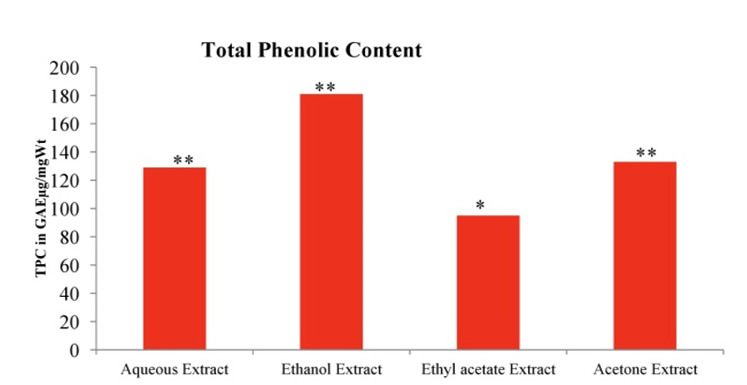
Totoal phenolic content of different solvent extracts of *Asanadi gana*in standard equivalents values are mean± standard deviation of three replication. Set of bars with different letters are significantly different * (p< 0.05 and p< 0.01)

The consequences of the total phenolic content were expressed in µg of GAE per mg (µg GAE/mg). The ethanolic extract had the highest total phenolic content of (181 ± 5.5µg GAE/mg) while the ethyl acetate extract had the lowest amount (95 ±2.8 µg GAE/mg). The aqueous and acetone extracts were 129 ± 2.2 µg GAE/mg and 133 ± 2.5 µmicro;g GAE/mg respectively. There was no noteworthy distinction between in TPC the aqueous and acetone extracts (P > 0.05) Figure 1.

## Total Flavonoid content (TFC):

**Figure 2:**
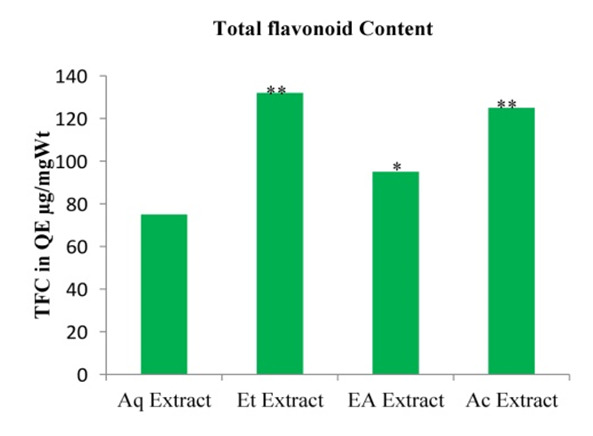
Totoal flavonoid content of different solvent extracts of *Asanadi gana*in standard equivalents values are mean± standard deviation of three replication. Set of bars with different letters are significantly different * (p< 0.05 and p< 0.01)

The total flavonoids content of the extracts was resolved with reference to the standard quercetin and expressed as its quercetin equivalent (µg QE/mg). The result of the assessment likewise demonstrated the ethanol extract to be significantly higher (132 ± 5.50 µg QE/mg) than the rest of solvent extract (P < 0.05). Acetone, ethyl acetate, and aqueous extracts had TFC values of 125 ± 2.80µg QE/mg, 95± 3.80µg QE/mg and 75 ± 2.80µg QE/mg respectively. All the solvent extracts were essentially not the same as one another (P < 0.05) Figure 2

## Total Tannin content (TTC):

**Figure 3:**
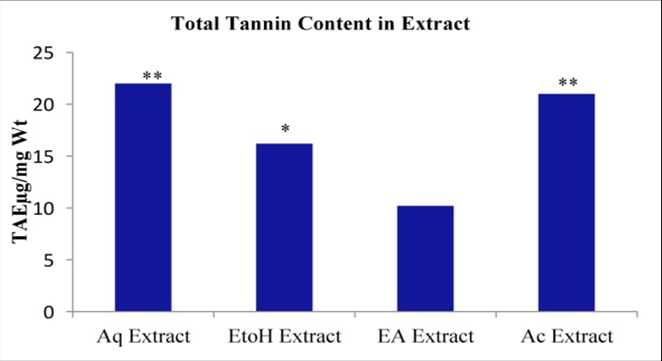
Totoal tannin content of different solvent extracts of *Asanadi gana*in standard equivalents values are mean± standard deviation of three replication. Set of bars with different letters are significantly different * (p< 0.05 and p< 0.01)

The total Tannin content of the extracts was resolved with reference to the standard Tannic acid and expressed as its Tannic acid equivalent (µg TAE/mg). The result of the evaluation showed TTC in the acetone and aqueous extract to be significantly higher (21± 1.8µg TAE/mg) and (22± 1.6µg TAE/mg) than the rest solvent extracts (P < 0.05). Ethanol and ethyl acetate extracts had TTC values 16.2± 2.1µgTAE/mg and 10± 2.0 µg TAE/mg respectively. Both solvent extracts were significantly different from each other (P < 0.05) Figure 3.

*Asanadi gana*which shows the maximum phenolic content with high antioxidant property is a combination of twenty-three herbs as shown in Table 2. It is a classical Ayurvedic combination detailed in the context of *Ashtanga Samgraha* [14] and *Ashtanga Hridaya* [15]. The combination has a broad spectrum of activity ranging from *Shwitra* (vitiligo), *Kushtha* (Chronic skin diseases), *Kaphaja-Vikara* (Inflammation diseases), *Krimi* (Infectious diseases), prameha (Obesity) and *Medodosha* (dyslipidemia). In clinical practice, it is used that in conditions like skin disorders, obesity, and anemia, whereas oxidative stress causes or aggravates the disease process [16], *Asanadi gana*is very effective to countercurrent the oxidative mechanism. The combination of *Asanadi gana*has many potent vatahara herbs (Calming Herbs to Balance Your Vata Dosha) which are also antioxidants like *Pterocarpus santalinus*, *Barberis aristata*, *Borassus flabellifer* and hypoglycemic like *Pterocarpus marsupium* , *Terminalia arjuna* , *Acacia catechu* Wild etc, The antioxidant and hypoglycemic effect of the combination in the treatment and management of madhumega (diabetes).

*Asanadi gana*extracts possessing moderate amount of flavonoids are very efficient scavengers of free radicals [17] because of their molecular structures, which consist of hydroxylated phenyl, hetrocyclic ring. The phenolic group in flavonoids scavenge both oxygen and nitrogen specious radicals through sharing of mobile hydrogen and also induce anti-oxidant enzymes genomes [18]. The unsaturated double bonds in carbon of flavonoids and isoflavonoids possess sharing of electrons to water insoluble free radicals with and inactivating superoxide anions, oxygen lipid peroxide radicals, and/or stabilizing free radicals involved in the oxidative process by hydrogenation or complexing with oxidant species [19]. Therefore, flavonoids act as chain braking antioxidant. OH• removal by flavonoids and isoflavonoids displayed a considerable antioxidant activity and may be capable of inhibiting cell damage caused by that radical and significantly decreasing the production of nitrite [19], It is believed that oxidative stress plays an important role in the development of vascular complications in diabetes particularly type-2 diabetes [20]. *Asanadi gana*poly herbal formulation formulated from many potent vatahara herbs like *Butea monosperma*, *Ougenia oojeinensis*, *Betula utilis*, *Acacia catechu* and *Anogeissus latifolia* rich source of flavonoids are very efficient scavengers of free radicals in management of diabetes.

Tannins are polyphenolic compounds that are present in various parts of plants [21]. They are available generally in the stem bark of trees which help to keep the plant cell free from microorganisms and parasites and few investigations affirmed that the tannins display hostile to oxidant, against microbial and calming properties [22]. *Asanadi gana*poly herbal formulation formulated from bark of following herbal plants *Pterocarpus marsupium*, *Holorrhena antidysentric* and *Vateria indica* is the source of tannin content in extracts. The potent ingredient of *Asanadi gana*is bark of asana (Pterocarpus marsupium) traditionally used for Diuretic, cholera, dysentery, stomachache, tongue diseases and the main pharmacological role of plant is antidiarrheal and antimicrobial activities due to presence of the active major phytoconstituent are tannins [21] and strong inhibitory activity with αalpha;- amylase and α- glycosidase [33].

## *Asanadi gana*demonstrated strong anti-oxidant activity:

## Total Antioxidant Capacity:

**Figure 4:**
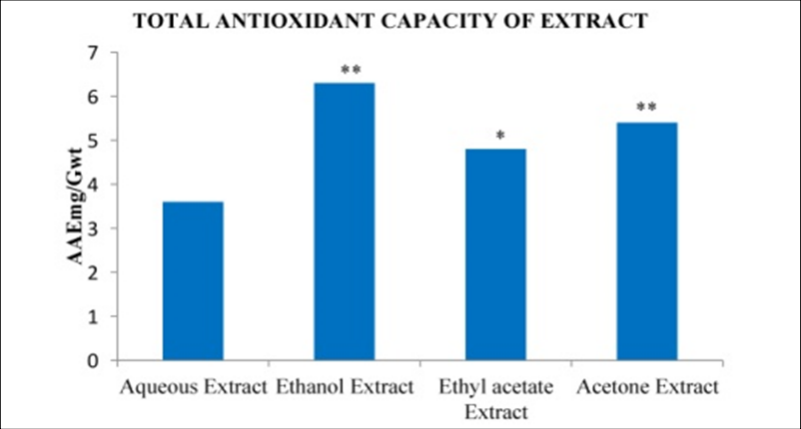
Ferric reducing antioxidant potential (FRAP) of different solvent extracts of *Asanadi gana*in standard equivalents values are mean± standard deviation of three replication. Set of bars with different letters are significantly different * (p< 0.05 and p< 0.01)

**Figure 5:**
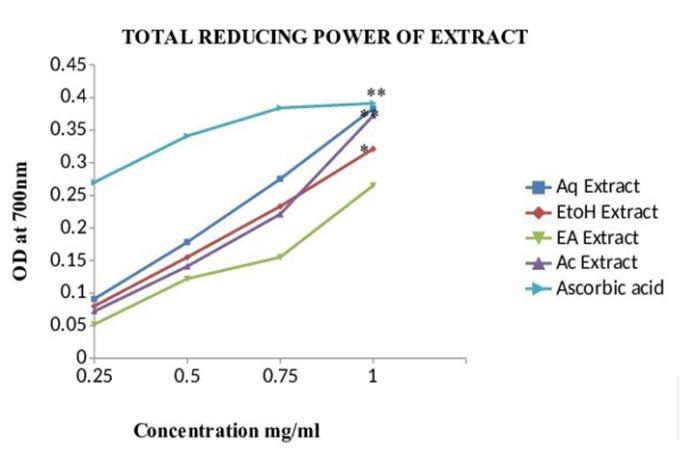
Plot: Ferric reducing antioxidant potential (FRAP) of different solvent extracts of *Asanadi gana*in standard equivalents values are mean± standard deviation of three replication. Set of bars with different letters are significantly different * (p< 0.05 and p< 0.01)

The total antioxidant potential is a relevant tool for investigating the relationship between dietary antioxidants and pathogenesis induced by the oxidative stress. In the present study, ferric ion reducing antioxidant power (FRAP) of different extracts of the polyherbal formulation *Asanadi gana*have been investigated. As shown in Figure 4, Figure 5: results were expressed in mg of AAE per gm (mg AAE/gm). The ethanolic extract had the highest total antioxidant potential (6.3±0.25mg AAE/gm) while the aqueous extract had the lowest amount (3.6±0. 25mg AAE/gm).T he acetone and ethyl acetate extracts were (5.4±0.1425mg AAE/gm) and 4.8±0.18mg AAE/gm) respectively. There was significant difference between the acetone and ethyl acetate extracts (P > 0.05). The term total antioxidant capacity, or TAC, emerged in an attempt to unify the concept. It was defined as the "cumulative action of all the antioxidants present in plants, thereby providing an integrated metric rather than the simple sum of measured antioxidants" in a review by nutritionists [23]. In the Ayurvedic literature "Sarangdhar Samhita" has highlighted the concept of poly-herbalism in this ancient medical system. This key traditional therapeutic herbal strategy exploits the combining of several medicinal herbs to achieve extra therapeutic effectiveness through cumulative action of antioxidants. The poly-herbalism concept in *Asanadi gana*is known to possess different variety of antioxidant potentials demonstrated by increased ferric ion reducing antioxidant power (FRAP).

## Total Reducing Power Assay:

**Figure 6:**
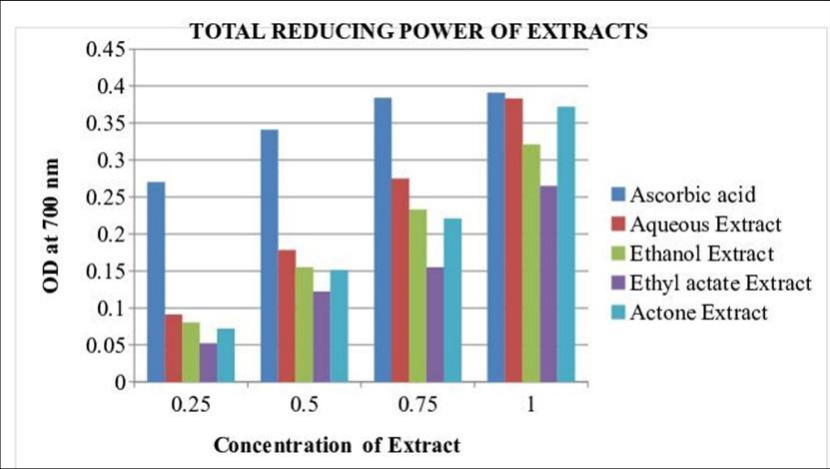
Bar Chart: Ferric reducing antioxidant potential (FRAP) of different solvent extracts of *Asanadi gana*in standard equivalents values are mean± standard deviation of three replication. Set of bars with different letters are significantly different * (p< 0.05 and p< 0.01)

The reducing power activity of the extract could serve as a significant indicator of the antioxidant potential. In the present study, the ability of the extract to transform Fe+3 to Fe+2.Compounds with reducing power indicate that they are electron donors and can reduce the oxidized intermediates of lipid peroxidation processes [24]. Table 3: and Figure 6: shown TRP values of different extract in different concentration expressed in Absorbance at 700nm. Ethanol extract have significant high absorbance (0.321±0.002) when compared to ascorbic acid standard (P<0.001). The reducing capacity of antioxidant is due to their electron transfer property such as polyphenols and flavonoids. Many studies demonstrated that the plants extract possess a strong reducing capacity. In other hand, many researchers has been widely reported the between polyphenol structure and their and ferric reducing capacity [25].

## DPPH Radical Scavenging Activity:

**Figure 7:**
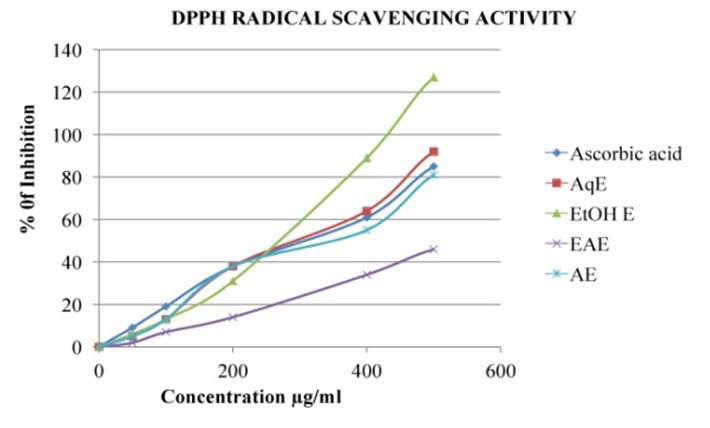
DPPH Radical Scavenging Activity of different solvent extracts of *Asanadi gana*in standard equivalents values are mean± standard deviation of three replication. Set of bars with different letters are significantly different * (p< 0.05 and p< 0.01)

**Figure 8:**
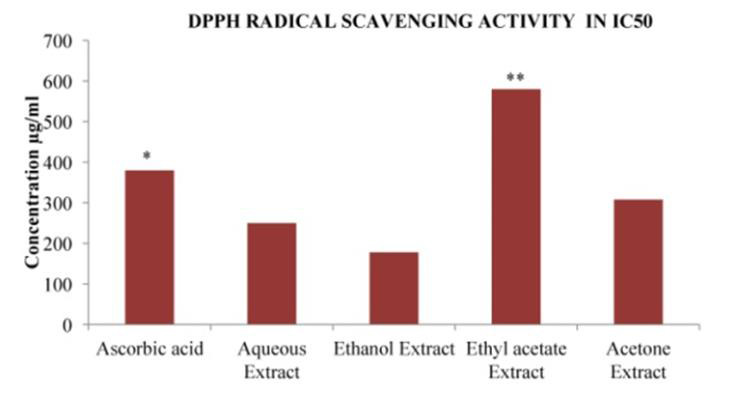
DPPH Radical Scavenging Activity in IC50of different solvent extracts of *Asanadi gana*in standard equivalents values are mean± standard deviation of three replication. Set of bars with different letters are significantly different * (p< 0.05 and p< 0.01)

One of the free radicals frequently used to assess a compound's or a plant extract's potential to scavenge radicals is DPPH. The parameter IC_50_, is used for the interpretation of the results from the DPPH method and is defined as the concentration of substrate that causes 50% loss of the DPPH activity [26]. In the present study, The DPPH radical activity in % of inhibition and IC50 of *Asanadi gana*different solvent extracts in comparison to known antioxidants (Ascorbic acid) and their respective concentrations were presented in Figure 7,Figure 8 and Table 4, respectively. The Organic radicals scavenging activity of all the solvent extracts and standard drug increased with increase in concentration range.

DPPH Radical Scavenging activity IC50 values of the different solvent extracts of *Asanadi gana*in different concentration Values are mean ± standard deviation of three replications. Set of bars with different letters are significantly different *(P < 0.05) and **(P < 0.01)x Ethanol extract had higher most DPPH inhibitor activity in IC50 (178µg/ml±6.87). The Increasing scavenging activity of the extracts and the standard drugs based on the IC was in the order; Vitamin C < acetone< aqueous < (Table 4).Different *in-vitro *methods were employed to determine the effect of antioxidant in plant extract. replication. Set of bars with different letters are significantly different * (p< 0.05 and p< 0.01)

DPPH radical scavenging method only model Antioxidant reaction with an organic Radical. *Asanadi gana*had higher constituents is Phenolic compounds(181 ± 5.5µg GAE/mg), Phenolic compounds are hydrogen offering antioxidants[27], thus higher radical scavenging activity of hydro-alcoholic extract may be attributed to higher amount of hydrogen donating phenolic antioxidants in ethanol extract.

## In Vitro Anti- Diabetic activity:

## Effect on α-amylase (EC 3.2.1.1) and α- Glucosidase (EC 3.2.1.21) activity:

**Figure 9:**
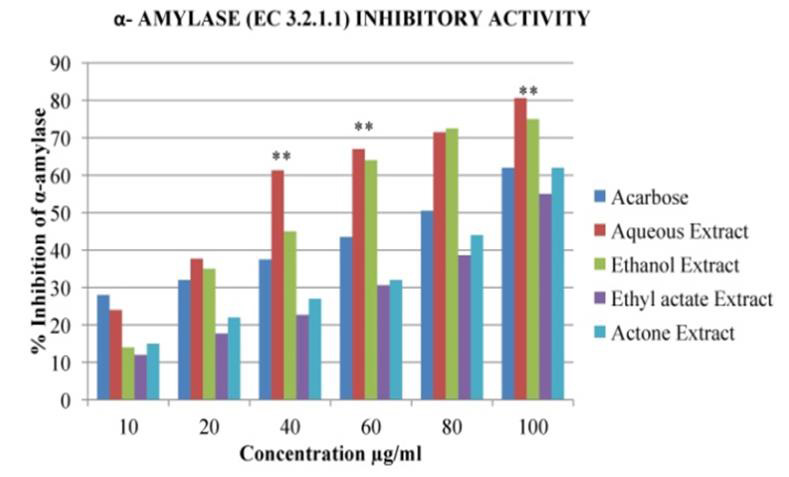
α - Amylase (EC.3.2.11) inhibitory Activity % inhibition values of different solvent extracts of *Asanadi gana*in standard equivalents values are mean± standard deviation of three replication. Set of bars with different letters are significantly different * (p< 0.05 and p< 0.01)

**Figure 10:**
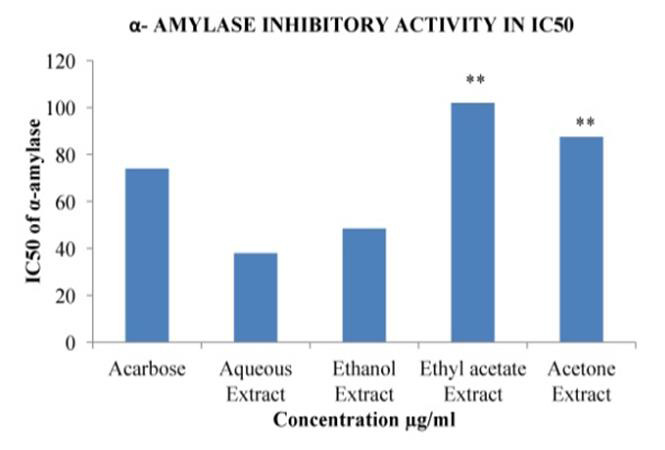
α - Amylase (EC.3.2.11) inhibitory Activity in IC50 values of different solvent extracts of *Asanadi gana*in standard equivalents values are mean± standard deviation of three replication. Set of bars with different letters are significantly different * (p< 0.05 and p< 0.01)

**Figure 11:**
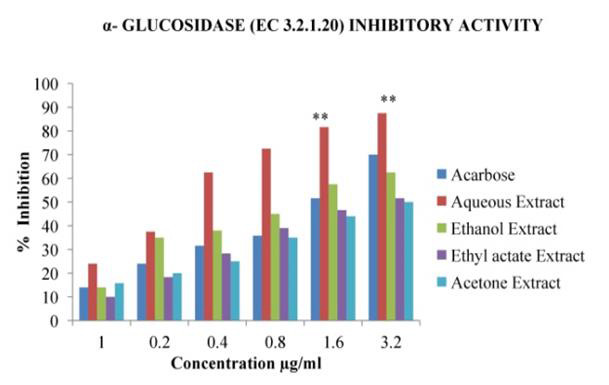
α - Glucosidase (EC.3.1.20) inhibitory Activity % inhibition values of different solvent extracts of *Asanadi gana*in standard equivalents values are mean± standard deviation of three replication. Set of bars with different letters are significantly different * (p< 0.05 and p< 0.01)

**Figure 12:**
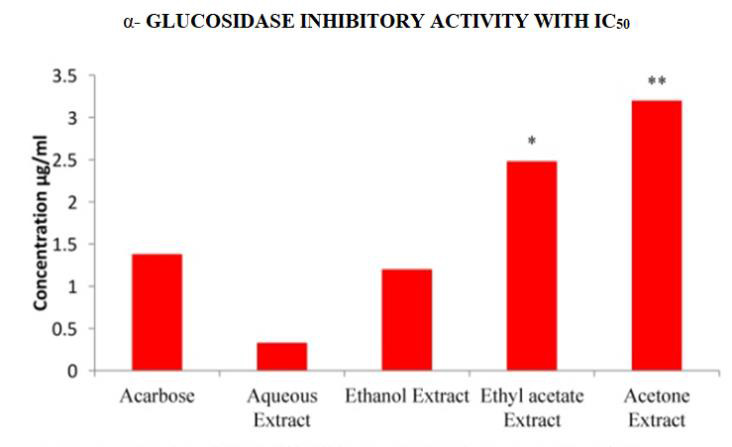
α - Glucosidase (EC.3.1.20) half inhibitory Concentration IC50inhibitory Activity in IC50 values of different solvent extracts of *Asanadi gana*in standard equivalents values are mean± standard deviation of three replication. Set of bars with different letters are significantly different * (p< 0.05 and p< 0.01)

The Ayurvedic literature *Charaka Samhita* and *Sushruta Samhita* gives a source of plants to treat different disease and plants hold definite guarantees within the management of diabetes mellitus [18,28]. In the present study, different solvent extracts of *Asanadi gana*polyherbal formulation were evaluated for their effect on alpha-amylase and alpha-glucosidase enzymes using *in-vitro *assays. Porcine pancreatic alpha-Amylase (PPA) is closely related to human alpha-Amylase [29]. Hence PPA was used to evaluate inhibitory activity of *Asanadi gana*extracts with starch as the substrate. *Asanadi gana*extracts inhibited α- amylase and α- glucosidase activity in dose dependent manner with reference standard, Acarbose expressed in percent inhibition and 50% inhibition of enzyme. Aqueous extract showed the highest α- amylase inhibitory activity and IC50, value of 38± 1.65µg/ml and 0.33±0.02µg respectively (Figure 9). Also, it has inhibited α- amylase more potently (P<0.05) than the standard Acarbose drug 74±1.96µg and 1.38±0.15µg respectively. The α-amylase inhibitory activity of the extracts and the standard drug based on the IC50 was in the order; aqueous > Acarbose < ethanol < acetone < ethyl acetate (Figure 10). The α-glucosidase inhibitory activity of the extracts and the standard drugs based on the IC50 was in the order; aqueous > ethanol > Acarbose < acetone < ethyl acetate (Figure 11Figure 11,Figure 12). According to Mogale et al., 2011 [30], natural inhibitors from plants are reported to have lower inhibitory effect against alpha-amylase and stronger inhibitory activity against alpha-glucosidase and our study supports this finding. In this study polyherbal formulation has shown higher inhibitory activity of carbohydrate digestive enzyme when compared to single drug Acarbose, *Asanadi gana* from various high degree hypoglycemic medicinal herbs [31] under the reference of ancient Ayurvedic literature "Sarangdhar Samhita" for polyherbalism.

Using the Ayurvedic concept on Polyherbal formulations (PHFs) provide treatment of diseases in a holistic approach. The scientific advancement carries with it the improvement in Ayurvedic concept of PHF, through the study of various phytoconstituents and discovery of useful herbs combinations which work synergistically to produce desirable effect. In our study we investigated all valid information regarding antidiabetic potential of *Asanadi gana*PHF; it was found to be source of versatile phytosignatures which possess cumulative antioxidant and hypoglycemic activity. The total antioxidant activity of PHF is due to the combined activity of phytoconstituents. Compound in PHF have the electron sharing antioxidants like polyphenols and proton donating antioxidants like flavonoids and tannins. The hypoglycemic activity of PHF is due to combined activity of the strong inhibiting activity of α-amylase (EC 3.2.1.1) and α- Glucosidase (EC 3.2.1.21) enzymes.

The role of oxidative stress in diabetes and diabetic complications has been reported [32]. Antioxidants can scavenge free radicals and play important role in prevention of diabetes. The hypothesis of this study is that all the ingredients may act synergistically as a potential phyto therapeutical weapon like a double edged sword executively and free radical scavenging activity on one side and α-glycosidase activity on the other side. Multi target mode of action, high safety and tolerability of phytotherapeutics offer valuable preventive and therapeutic options in holistic diabetes management. This work has laid the blue print for the quality control that is expected to pave way for use of this formulation as a dietary supplement in DM.

## Conclusion:

In the present study, we conclude that the extracts of ayurvedic polyherbal formulations, particularly ethanol extract are a potential source of natural antioxidants and remarkable glycosidase inhibitory activity. Hence, *Asanadi gana*may be regarded as a promising natural and safe remedy for the prevention or delay of diabetic complications.

## Figures and Tables

**Table 1:** Qualitative Identification of primary phytochemicals in Asnadi gana extracts (AGE)

**S.No**	**Phytochemicals**	**Aqueous Extract**	**Ethanol Extract**	**Ethyl acetate Extract**	**Acetone Extract**
1	Terpenoids	-	+	+	+
2	Triterpenoids	+	+	-	-
3	Phenolics	+	+	+	+
4	Carbohydrate	+	+	+	+
5	Sterols	-	+	+	+
6	Tannins	+	+	-	-
7	Flavonoids	+	+	+	+
8	Protein	+	-	-	-
9	Glycosides	+	+	-	-
10	Alkaloids	+	+	-	-
11	Organic Acids	+	+	+	+
12	Saponins	+	+	-	-
+ present, - absent.

**Table 2:** Component of medicinal plants of *Asanadi gana*ayurvedic polyherbal formulation

**S. No**	**Name of Plant**
1	*Pterocarpus marsupium* Roxb.
2	*Ougenia oojeinensis* Roxb.
3	*Betula utilis*
4	*Terminalia arjuna* Roxb.
5	*Holoptelea integrifolia*
6	*Acacia catechu* Wild
7	*Acacia suma Buch.*
8	*Albizzia lebbeck Benth.*
9	*Dalbergia sissoo Roxb.*
10	*Gymnema sylvestre*
11	*Santalum album Linn.*
12	*Pterocarpus santalinus* Linn
13	*Barberis aristata* DC.
14	*Borassus flabellifer* Linn.
15	*Butea monosperma* Lam.
16	*Aquillaria agallocha Roxb.*
17	*Tectona grandis Linn.*
18	*Shorea robusta Gaertn.*
19	*Areca catechu Linn.*
20	*Anogeissus latifolia* wall.
21	Holorrhena antidysentrica Linn.
22	*Vateria indica* Linn.
23	Dipterocarpus turbinatus Geartn.

**Table 3:** Total Reducing Power Assay of *Asanadi gana*extracts: Absorbance at 700 nm at different concentration

**Extract**	**0.25mg/ml**	**0.5mg/ml**	**0.75mg/ml**	**1mg/ml**
Aqueous Extract	0.091±0.003	0.178±0.004	0.275±0.001	0.383±0.003**
Ethanol Extract	0.080±0.003	0.155±0.002	0.233±0.003	0.321±0.002*
Ethyl acetate Extract	0.052±0.004	0.122±0.004	0.155±0.004	0.265±0.004
Acetone Extract	0.072±0.004	0.151±0.002	0.221±0.004	0.372±0.004**
Ascorbic acid	0.27±0.009	0.341±0.004	0.384±0.005	0.391±0.003
Total Reducing Power (TRP) of the different solvent extracts of *Asanadi gana*in different concentration Values are mean ± standard deviation of three replications. *(P < 0.05) and **(P < 0.01)

**Table 4:** DPPH IC50 value of *Asanadi gana*extracts:

**S.No**	**Name of Extract**	**IC50 value**
1	Ascorbic acid	380µg/ml±11.5
2	Aqueous Extract	250µg/ml±8.22**
3	Ethanol Extract	178µg/ml±6.87 **
4	Ethyl acetate Extract	580µg/ml±17.3
5	Acetone Extract	308µg/ml±12.4*
